# Reconfigurable superconducting vortex pinning potential for magnetic disks in hybrid structures

**DOI:** 10.1038/srep45182

**Published:** 2017-03-24

**Authors:** Estefani Marchiori, Peter J. Curran, Jangyong Kim, Nathan Satchell, Gavin Burnell, Simon J. Bending

**Affiliations:** 1 Department of Physics, University of Bath, Bath, BA2 7AY, United Kingdom; 2 School of Physics and Astronomy, University of Leeds, Leeds, LS2 9JT, United Kingdom; 3 Center for Biomolecular Nanotechnologies, Italian Institute of Technology, Genova, 16163, Italy; 4 ISIS Neutron and Muon Source, Didcot, OX11 0QX, United Kingdom

## Abstract

High resolution scanning Hall probe microscopy has been used to directly visualise the superconducting vortex behavior in hybrid structures consisting of a square array of micrometer-sized Py ferromagnetic disks covered by a superconducting Nb thin film. At remanence the disks exist in almost fully flux-closed magnetic vortex states, but the observed cloverleaf-like stray fields indicate the presence of weak in-plane anisotropy. Micromagnetic simulations suggest that the most likely origin is an unintentional shape anisotropy. We have studied the pinning of added free superconducting vortices as a function of the magnetisation state of the disks, and identified a range of different phenomena arising from competing energy contributions. We have also observed clear differences in the pinning landscape when the superconductor and the ferromagnet are electron ically coupled or insulated by a thin dielectric layer, with an indication of non-trivial vortex-vortex interactions. We demonstrate a complete reconfiguration of the vortex pinning potential when the magnetisation of the disks evolves from the vortex-like state to an onion-like one under an in-plane magnetic field. Our results are in good qualitative agreement with theoretical predictions and could form the basis of novel superconducting devices based on reconfigurable vortex pinning sites.

The antagonistic nature of superconducting and ferromagnetic materials gives rise to many fascinating phenomena when both are placed in intimate contact. Unlike ferromagnetic superconductors, where superconductivity and ferromagnetism coexist in the same region of space, in artificial hybrid structures the interaction is limited to short distances around the interfaces between the subsystems^1^. Despite recent advances in our understanding of the physics of hybrid superconductor-ferromagnet structures, including the phenomena of domain-wall superconductivity^2^ and field-induced superconductivity^3^, the systematic control of superconducting vortices using nanofabricated pinning landscapes remains a major challenge for both fundamental science and applications in high current conductors. The importance of the size, spacing and material properties of magnetic pinning centres^4^,^5^ has mainly been explored by indirect measurements^6^,^7^. Here we demonstrate by direct vortex imaging that the controllable magnetic states of permalloy (Py) ferromagnetic disks can be exploited to sensitively tune the pinning landscape in hybrid samples. The interaction between a superconducting vortex line and similar ferromagnetic structures has been previously studied theoretically^8^,^9^ but the validity of these results remains to be experimentally verified. There are several possible sources of pinning in our structures such as the local suppression of superconductivity due to exchange-mediated Cooper pair breaking, corrugations in the topography of the Nb film due to the underlying Py disks and local weakening of the superconducting order parameter by the stray fields of the disks^10^. Here we describe a systematic study that allows us to separate different contributions of the magnetic and electronic interactions between the subsystems. In addition to the enhanced vortex pinning that arises in hybrids, the magnetic structure of the ferromagnet can play a much deeper role. In the last decade an intriguing long range proximity effect has been reported^11^,^12^ whereby non-collinear magnetic moments, such as those in the ferromagnetic structures studied here, lead to spin triplet pair correlations and superconducting currents that are not supressed by the local exchange field on long length scales^13^.

## Experimental Method

We have studied the interplay between superconducting and magnetic vortices in hybrid structures composed of a 4 *μ*m period square array of 1 *μ*m diameter, 50 nm high ferromagnetic Py (Ni_81_Fe_19_) disks produced by electron-beam lithography and magnetron sputter deposition onto a thermally oxidized Si substrate (90 nm SiO_2_). The ferromagnetic disks exhibit a weak magnetic anisotropy which results in a cloverleaf-like stray magnetic field distribution, somewhat distinct from the flux-closed magnetic vortex structures expected in disks of this size and shape^14^,^15^,^16^. A 100 nm superconducting Nb thin film has been grown by sputter deposition directly on top of the array, and the out-of-plane stray magnetic fields of the Py disks are expected to induce and pin superconducting vortices in it. Finally Ge(20 nm)/Au(50 nm) was thermally evaporated over the entire sample to facilitate the approach of the tunnelling tip integrated into the Hall sensor prior to magnetic imaging. When the ferromagnet is in direct contact with the superconductor, the superconducting order parameter is locally suppressed due to exchange-mediated pair breaking^17^. In order to supress this, a second sample with a thin alumina (Al_2_O_3_) dielectric layer between the disk array and the Niobium film has been realized by O_2_ plasma oxidation of a 1.6 nm thick sputtered Al film. Scanning Hall probe microscopy (SHPM) has been used to directly image the stray fields of the Py disks and the behaviour of vortices nucleating at T < T_*c*_(Nb) with high spatial resolution and sensitivity^18^. The SHPM used is a modified commercial low temperature scanning tunnelling microscope (STM) where the tunnelling tip has been replaced by a custom-fabricated GaAs chip. The Hall probe is patterned in the two-dimensional electron gas of a GaAs/AlGaAs heterostructure, defined by the intersection of two 500 nm wide wires situated ~5 *μ*m from the Au coated corner of a deep mesa etch that acts as an integrated STM tip. The Hall probe is mounted at an angle of 1°–2° with respect to the sample plane, with the STM tip being the closest point to the sample surface. In practice the sample is first approached towards the sensor until tunnelling is established and then retracted ~100 nm for rapid ‘flying mode’ scanning. Hence the active Hall probe is ~200–300 nm above the sample during scans^19^. In this way quantitative maps of the z-component of magnetic induction can be captured non-invasively, in contrast to other imaging techniques such as low temperature magnetic force microscopy (LT-MFM) where the magnetic tip can potentially nucleate or drag superconducting vortices^20^,^21^.

### Data accession

All data discussed in this manuscript can be found at https://doi.org/10.15125/BATH-00340.

## Results

**Figure 1:**
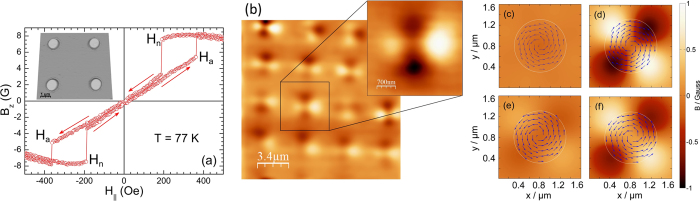
(**a**) Local magnetic induction as a function of in-plane field measured with the Hall probe parked near the centre of a Py disk. Inset shows an AFM image of the array of 1 *μ*m disks. (**b**) SHPM image of the Py disk array at 77 K with a colorscale of 0.8 G. Also shown is an expanded view of one 1 *μ*m Py disk. OOMMF simulations of the perpendicular magnetic stray field component 500 nm above (**c**) a 1 *μ*m diameter circular disk without anisotropy, (**d**) an elliptical disk with major axis (y-axis) of 1.04 *μ*m and minor axis (x-axis) of 1 *μ*m without magnetocrystalline anisotropy, (**e**) a 1 *μ*m diameter circular disk with uniaxial anisotropy constant *K*_1_ = 500 J/m^3^ and (f) a 1 *μ*m diameter circular disk with uniaxial anisotropy constant *K*_1_ = 1.5 × 10^3^ J/m^3^. The blue arrows represent the simulated magnetisation on the structure surface.

The Nb film in direct contact with the ferromagnet disks has a critical temperature *T*_*c*_ = 7.10 ± 0.05 K, while the sample with a dielectric spacer has a *T*_*c*_ = 7.20 ± 0.05 K, somewhat lower than the bulk critical temperature of 9.22 K^22^ due to the reduced film thickness and the non-optimised sputter deposition conditions used. An atomic force microscopy (AFM) image of the disk array is shown as an inset to Fig. 1(a), confirming the dimensions and height of the array of Py disks of the hybrid structure.

Figure 1(a) shows a measurement of the local magnetic induction as a function of in-plane field with the Hall sensor parked near the centre of a Py disk at 77 K. Due to the shallow tilt angle of the Hall probe we have measured the in-plane component of the magnetic induction which exhibits the classic form for a flux-closed magnetic vortex, showing the signature of vortex annihilation at ±362 Oe, and vortex nucleation at ±190 Oe. Figure 1(b) shows an SHPM image of a region near the centre of an array at 77 K (T ≫ T_*c*_) in zero applied field revealing that the remanent magnetisation state of the Py disks has cloverleaf-like stray fields. An ideal magnetic vortex would be fully flux-closed except for a very narrow core at the centre of the disk where the magnetisation points out-of-plane. In Fig. 1(b) three disks with reversed vorticity can be seen, the direction of the magnetisation rotation is statistically distributed between both orientations and cannot be controlled by in-plane magnetic fields^15^. We believe that the ‘cloverleaf’ fields seen here arise from very slight out-of-plane canting of the moments due to weak uniaxial in-plane anisotropy, analogous to structures seen in elliptical Py structures with in-plane shape anisotropy^23^.

The origin of the observed magnetisation state has been investigated by micromagnetic simulation using the OOMMF code^24^, which is based on the deterministic Landau-Lifshitz-Gilbert equation. Typical parameters for Py have been used: exchange constant *A* = 1.3 × 10^−11^ J/m and saturation magnetisation *M*_*s*_ = 8 × 10^5^ A/m. The spatial distribution of the perpendicular magnetic stray field component 500 nm above the centre of the disk is shown in Fig. 1(c–f) approximately simulating the Hall probe signal. Figure 1(c) illustrates the result for an ideal magnetic vortex with perfect circular shape and no magnetocrystalline anisotropy, when just weak fields around the central core are observed. Two possible origins have been considered for the cloverleaf-like stray fields. Figure 1(d) shows results for a slightly elliptical-shaped disk with semi-axes of 0.5 *μ*m (along x) and 0.52 *μ*m (along y). The second possibility that is considered is the introduction of a uniaxial magnetocrystalline anisotropy along the y-axis during Py film growth in the magnetic field from the special compact design of the magnetron sputtering head. Two anisotropy constants have been considered *K*_1_ = 500 J/m^3^ which is approximately the highest value reported for magnetron sputtered Py^25^ shown in Fig. 1(e), and *K*_1_ = 1.5 × 10^3^ J/m^3^ which represents an unrealistically high value for Py, shown in Fig. 1(f). One can see that the simulation for the weakly elliptical disk shows a stray field intensity and profile that is very similar to that measured with the SHPM. In contrast the simulation with a typical level of growth field-induced uniaxial anisotropy exhibits much lower fields than seen experimentally, and only matches experiment for the unrealistically large values of K_1_ used in Fig. 1(f). This suggests that the ‘cloverleaf’ field distributions arise primarily from systematic elliptic distortions of the disk introduced unintentionally during lithography or Py deposition.

After cooling samples to *T* = 5 K (<*T*_*c*_), the magnetic stray field of the disks that threads the Nb film must now satisfy fluxoid quantization. Depending on the stray field intensity, it will either be screened or formed into quantised superconducting vortices. The penetration depth for similar 100 nm sputtered Nb films has been reported to be *λ*(0) = 120 ± 10 nm^26^ and an effective Pearl penetration depth of Λ = *λ*^2^/*d* ~ 140 ± 20 nm is expected for thin films in perpendicular fields in the limit *d* ≪ *λ*^27^,^28^. Therefore, the disks are many times larger than the penetration depth and each lobe of the cloverleaf-like stray fields can be quantised independently.

**Figure 2:**
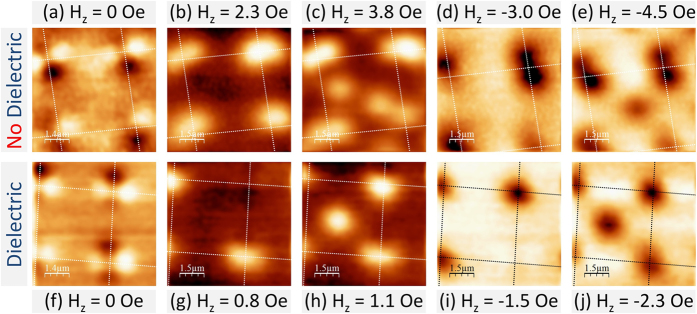
(**a**–**e**) SHPM images of the hybrid structure without a dielectric layer (**a**) at T = 7.5 K, and (**b**–**e**) after field-cooling to 5 K in the indicated magnetic fields. (**f**–**j**) SHPM images of the hybrid structure with a dielectric layer (**f**) at T = 7.5 K, and (**g**–**j**) after field-cooling to 5 K in the indicated magnetic fields.

In order to shed further light on these issues SHPM scans have been performed in small out-of-plane magnetic fields, H_*z*_. By simultaneously imaging the cloverleaf-like stray magnetic fields due to the Py disks, and vortices nucleating above them, the relationship between them could be determined. Figure 2 shows SHPM images of central regions of both samples. Figure 2(a) and (f) were acquired above T_*c*_ at 7.5 K in H_*z*_ = 0 and clearly show the cloverleaf-like stray fields. All other images were obtained after field-cooling to 5 K in the indicated out-of-plane field ((a–e) sample without dielectric layer; (f–j) sample with a dielectric layer). White or black dotted lines have been superimposed on the scans to indicate the centres of Py disks where these lines intersect.

In order to identify vortex locations and magnitudes more precisely a careful line profile analysis and sequential image subtraction have been performed. Vortices that are less than ~500 nm apart cannot be resolved due to the finite spatial resolution of the Hall probe. In such cases the number of vortices has been estimated by integrating the flux associated with the object. These indicate that two vortices (antivortices) nucleate on top of both bright (dark) lobes of a disk in the sample without the intermediate dielectric layer. The large approximately oval white structures in Fig. 2(b) and (c) contain two vortices side by side. When the sign of the applied field is reversed two black antivortices can be seen on top of the black ‘cloverleaf’ lobes in Fig. 2(d) and (e). In contrast only one vortex (antivortex) nucleates over the centre of the disk in the sample with an intermediate dielectric layer as shown in Fig. 2(g–j).

**Figure 3:**
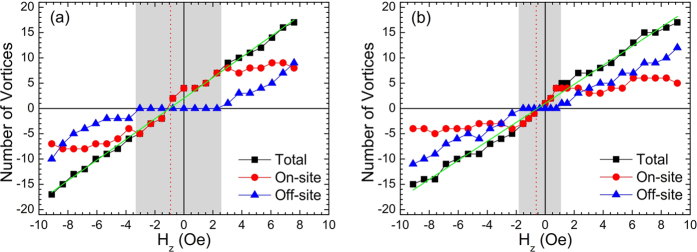
Number of vortices nucleated in the field of view as a function of perpendicular cooling field, (**a**) without intermediate dielectric layer and (**b**) with a dielectric layer. Circles and triangles relate to vortices nucleating above disks or between disks respectively. Black squares indicate the total number of vortices. The dark green fitting line indicates a linear nucleation trend.

As the cooling field is increased the pinning sites above the disks eventually become saturated and interstitial vortices (antivortices) start to be nucleated, as shown in Fig. 2(c,e) for the sample without the dielectric layer and in Fig. 2(h,j) with it. In the latter case interstitials nucleate at a lower field due to the overall weaker pinning potential. This is clearly shown in Fig. 3(a,b) which plot the cooling-field dependencies of the on-site (circles) and off-site (triangles) vortices in the two samples and the gray shading indicates the regions where all vortices were pinned on top of the disks. The black squares indicate the total number of vortices nucleated inside the scan area, and depends approximately linearly on applied magnetic field in both cases. Note that the intercept with the field axis where the number of vortices drops to zero (indicated by a vertical dotted line) passes to the left of the origin due to the earth’s magnetic field at the sample space.

**Figure 4:**
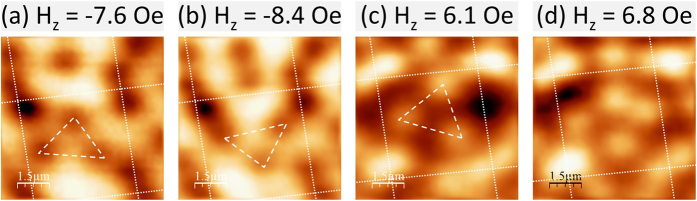
SHPM images near the centre of a sample without the dielectric layer after field-cooling to 5 K in the indicated magnetic fields, showing an approximately triangular interstitial vortex (antivortex) alignment.

At the highest fields shown in Fig. 2(e) and (j) a single interstitial vortex can be seen close to the centre of a unit cell, stabilized by repulsive interactions with pinned on-site vortices. At still higher fields more interstitial vortices nucleate and organize under their mutually repulsive interactions. Figure 4 shows that the competition between interactions with static on-site vortices and other mobile off-site vortices leads to approximately triangular arrangements that rotate as the cooling field is increased (c.f., Fig. 4(a–d)). In both cases the rotation is seen to be driven by repulsion from a new vortex nucleating near the perimeter of the unit cell.

**Figure 5:**
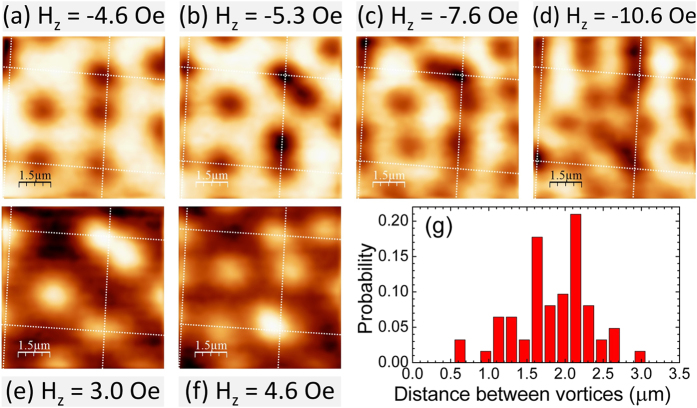
(**a**–**f**) SHPM images of the system with a dielectric layer after field-cooling to 5 K in the indicated fields, and (**g**) histogram of the nearest neighbour vortex spacing in (**d**).

Figure 5 shows images of the sample with the dielectric layer at high positive and negative fields. Surprisingly we observe pairs of closely-spaced vortices formed by one interstitial and one on-site vortex. As the cooling-field is increased the position of the off-site vortex changes in response to the nucleation/movement of nearby interstitials (c.f., the pair of antivortices in the top right hand corner of Fig. 5(a,b) and the pair of vortices at the same location of Fig. 5(e,f)). In Fig. 5(c) we find that a third antivortex has been added to the original pair on the upper right disk, suggesting an unexpected attractive interaction between on-site and nearby interstitial fluxons. It is possible that the topographic corrugation of the Nb film due to the underlying disk perturbs supercurrent trajectories and partially supresses the repulsive forces between on-site and off-site vortices. The coupled interstitial vortices show no preferential positions around the disk, indicating no direct interaction with the magnetic stray field lobes of the disks.

Vortices of the same polarity normally repel each other in such strongly type II materials and the presence of competing attractive forces is not expected. Similar pair formation has been observed by Kramer *et al*.^29^, but in a system of Co/Pt multilayer disks with out-of-plane magnetisation, where spontaneous vortex-antivortex pairs were expected. Figure 5(g) shows a histogram of the nearest neighbour antivortex distances determined by Delaunay triangulation of the vortex image at H_*z*_ = −10.6 Oe (Fig. 5(d)). This appears to show a bimodal distribution reflecting the clustering of interstitial antivortices around the Py disks with more widely spaced fluxons near the middle of the unit cell. There also appears to be a tendency for connected chains of antivortices to form between Py disks as seen in Fig. 5(d).

**Figure 6:**
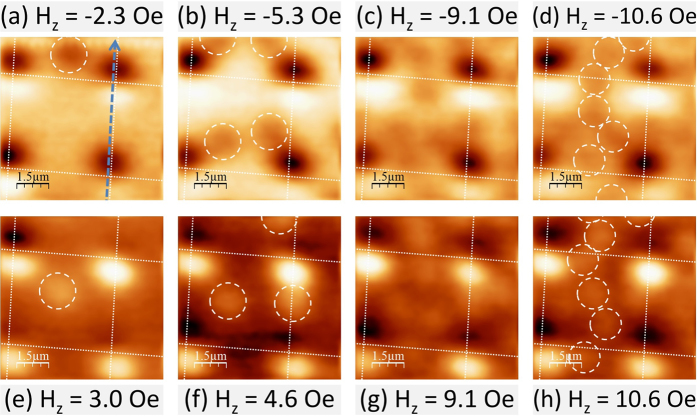
SHPM images of the system with a dielectric layer after field-cooling to 5 K in H_||_ = 50 Oe and the indicated out-of-plane fields.

A constant in-plane magnetic field, H_||_ = 50 Oe, has also been applied to the samples to produce a so-called ‘onion’ magnetic dipole state. In this state the Py disks are not fully saturated as the vortex core has not been annihilated, it is probably pinned near the edge of the disk. The opposite magnetic stray fields on the poles of each disk are expected to nucleate and pin vortices and antivortices. In practice both arrays exhibited very similar behaviour as illustrated for the sample with an intermediate dielectric layer in Fig. 6. The number of vortices and antivortices spontaneously nucleated on the poles of the Py disks after field-cooling to 5 K has been estimated by analysing line scans across them.

In order to satisfy fluxoid quantisation, externally applied out-of-plane fields passing through the sample must either nucleate new vortices (antivortices) or annihilate existing antivortices (vortices). Both processes can break the vortex/antivortex symmetry of the poles of the Py disks.

**Figure 7:**
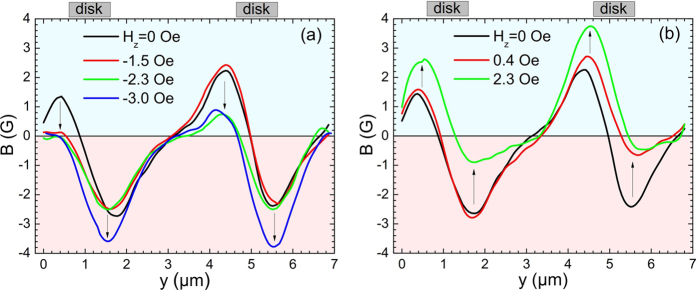
Line scans across two adjacent Py disks in the structure with a dielectric layer in an ‘onion’ magnetisation state (H_||_ = 50 Oe) revealing nucleation and annihilation events when an (**a**) negative and (**b**) positive out-of-plane field is applied. The dashed blue arrow on Fig. 6(a) indicates the orientation and position of the line scans.

Vortex nucleation or annihilation appears to evolve in a very complex way and the net flux associated with each disk does not vary smoothly with out-of-plane magnetic field. Figure 7 shows line scans across two adjacent disks (indicated by a dashed line in Fig. 6(a)) after field-cooling in negative (Fig. 7(a)) and positive (Fig. 7(b)) out-of-plane fields. An effective field of H_*eff*_ = −1 Oe at zero applied field has been estimated due to the sum of the contributions of the parasitic out-of-plane component of the electromagnet and the Earth’s magnetic field. For negative fields we first see vortex annihilation (green line; H_*z*_ = −2.3 Oe) followed by antivortex nucleation (blue line; H_*z*_ = −3.0 Oe). For positive fields we first see antivortex annihilation (red line; H_*z*_ = 0.4 Oe) followed by simultaneous antivortex annihilation and vortex nucleation (green line; H_*z*_ = 2.3 Oe). When one on-site vortex(antivortex) is annihilated the opposite on-site antivortex(vortex) appears to broaden as clearly seen in the line scan.

The interstitial vortices and antivortices visible in Fig. 6 have also nucleated nearest to poles of the same polarity, contrary to the expectation that they should be repelled from these. Upon further increasing the number of interstitial antivortices or vortices, as shown in Fig. 6(d) and (h), a zigzag-like structure can be seen between disks, apparently caused by competing repulsive and attractive interactions.

**Figure 8:**
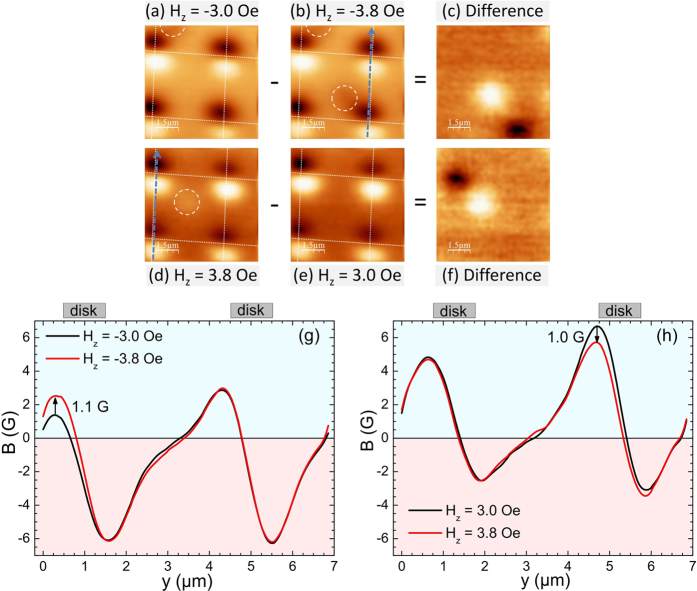
SHPM images at fixed H_||_ = 100 Oe after field-cooling to 5 K in out-of-plane field (**a**) H_*z*_ = −3.0 Oe, (**b**) H_*z*_ = −3.8 Oe, (**c**) Difference image (**a**)–(**b**), (**d**) H_*z*_ = 3.8 Oe, (**e**) H_*z*_ = 3.0 Oe, (**f**) Difference image (**d**)–(**e**). (**g**,**h**) Line scans across two adjacent disks indicated by the dashed blue arrows in (**a**,**d**) showing on-site antivortex annihilation in (**a**) and vortex annihilation in (**c**) respectively.

When the fixed in-plane magnetic field is increased to H_||_ = 100 Oe the system shows a similar vortex nucleation/annihilation behaviour, but a clear link between interstitial and on-site vortices can now be made. This shows a restored symmetry in comparison to the broken symmetry when half of the in-plane magnetic field is applied. Figure 8(a) shows the nucleation of an interstitial antivortex linked to the annihilation of an on-site antivortex as is clearly seen in the difference image (Fig. 8(c)) where the white spot represents a new antivortex and the black spot a missing antivortex. The same is seen in in Fig. 8(d,f) where an on-site vortex has been annihilated and an interstitial vortex nucleated. The line scans in Fig. 8(g,h) along the dashed lines shown in Fig. 8(a,d) confirm the annihilation of an on-site antivortex(vortex) when the field is increased. In both Fig. 8(g,h) we can associate a change in the peak dipole field of ~1.0 ± 0.1 G with a change in its population by one vortex(antivortex).

## Discussion

The presence of two vortices(antivortices) situated close to each other on top of a disk rather than the formation of a triangular Abrikosov lattice usually observed in Nb thin films^30^ suggests that a reasonably strong pinning potential is at play. We have estimated the pinning force (F_*p*_) required to balance the repulsive force (F_*r*_) between the two vortices in the top right hand corner of Fig. 2(d) which are 1.0 ± 0.1 *μ*m apart. Using an expression due to Pearl^27^ for the repulsive interaction between two vortices at a separation *r* in an infinite superconducting thin film of thickness d ≪ *λ*,


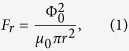


where Φ_0_ is the superconducting flux quantum, the repulsive force is estimated to be F_*r*_ = 1.1 ± 0.2 pN (pinning force per unit length, f_*r*_ = 11 ± 2 pN/*μ*m), approximately half the value found by Shapoval^31^ in a similar hybrid structure.

Even though the pinning potentials represented by the Py disks disturb the triangular vortex ordering expected for Nb thin films, it is interesting to compare the vortex spacing in the triangular interstitial vortex structures of Fig. 4 with the expectation for an Abrikosov vortex lattice.


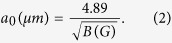


The estimated lattice parameter, *a*_0_, and the average measured distance between the (anti)vortices, *a*_*m*_, in this triangular structure has been compared for the effective magnetic fields applied of −7.5 Oe and 7.0 Oe in Fig. 4(b) and (c), respectively, where the effective field of −0.9 Oe at zero applied field has been estimated from Fig. 3(a). For Fig. 4(b) *a*_0_ = 1.78 *μ*m and *a*_*m*_ = 2.26 *μ*m and for Fig. 4(c) *a*_0_ = 1.85 *μ*m and *a*_*m*_ = 2.34 *μ*m. The systematic deviation reveals that the interstitial Abrikosov vortex lattice not only depends on the magnetic field but also on the attractive potential experienced by on-site vortices. Hence the vortex-vortex spacing and orientation of the interstitial triangular arrangements are controlled by the location of the on-site vortices which are in turn controlled by the magnetisation structure of the Py disks.

Vortices and antivortices have preferentially pinned in different locations in the two types of samples suggesting a qualitatively different pinning landscape. In the hybrid structure without an insulating layer the ferromagnetic disks and the superconducting layer are electronically coupled and the vortices experience a uniform pinning potential over the whole area of the disk due to exchange-mediated pair breaking. The cloverleaf-like stray fields of the Py disks lead to additional pair breaking and field polarity-dependent pinning. In contrast exchange-related pinning is absent from the sample with the intermediate dielectric layer, and only pinning due to the Py stray fields plays a role. Hence the pinning potential is much shallower and only a single vortex can be pinned on the disk, possibly centred on the core of the underlying magnetic vortex.

Existing theoretical papers^8^,^9^ assume an insulating layer between the superconducting and ferromagnetic films, thus we can only compare predictions with the hybrid system with an intermediate dielectric layer. Both papers use the London theory to describe the response of a thin superconducting film with a single ferromagnet nanostructure sitting on top. Although the models do not consider dipole-dipole interactions with other structures in an array, the in-plane dipole field of our disks in the vortex state and at saturation are estimated to be zero and *H*_*sat*_ ~ *m*/*a*^3^ ~ 6 *Oe*, respectively, where *a* is the distance to the nearest neighbour disk. This maximum field is very much smaller than typical magnetic vortex annihilation fields and should only represent a weak perturbation in our experimental system. Although our hybrid structures have somewhat larger characteristic dimensions than those considered in these papers, the qualitative vortex behaviour seems to be well captured. In ref. 8, the vortex pinning potential is calculated as a ‘rigid’ magnetic vortex core is displaced by an in-plane field, modifying the disk magnetisation. Even though spontaneous vortex-antivortex nucleation and vortex-vortex interactions are not considered, the predicted linear increase in pinning potential with in-plane field agrees qualitatively with the increasing strength of vortex pinning as we move from the vortex-like magnetisation state to the onion-like one. Interestingly, a minimum in the pinning potential located in a circle around the disk edge in the vortex state is predicted, which may possibly be related to the apparent attractive pairwise vortex interaction we observe at low in-plane fields (c.f., Fig. 5), though seems unlikely to explain similar observations at high in-plane fields. Reference 9 considers a bar with fixed in-plane magnetisation and studies the pinning of a new free vortex on one of the poles as a function of magnetisation and the presence of nucleated vortex-antivortex pairs. Since these authors show that the field profile of a square and a disk give rise to qualitatively similar magnet-vortex interactions, we have compared our results with the predicted threshold magnetisation necessary for the nucleation of vortex-antivortex pairs by a square magnetic nanostructure. Our predicted M/M_0_ ratio at saturation of 0.39, lies well above the range considered in Fig. 9 of ref. 9, but an extrapolation of the calculated data suggests that significantly more than two spontaneous vortex-antivortex pairs should form. In the partially magnetised onion-like state at H_||_ = 100 Oe we find a maximum of four vortices(antivortices) nucleated on each pole of the disk which is in qualitative agreement with this. In our hybrid system the polarity-dependent pinning of vortices as a function of out-of-plane field at H_||_ = 50 Oe exhibits a very complex history of vortex nucleation and annihilation events. This is also easy to understand in terms of Fig. 9 of ref. 9 which shows regions where free vortices are pinned alternately at the positive or negative poles of the ferromagnet as its magnetisation is increased, though does not explain why the behaviour in H_||_ = 100 Oe appears to become somewhat more predictable. There are several other features of our experiments that do not have a natural interpretation in existing theoretical models such as the pairwise attraction between on-site and off-site vortices (antivortices) at high in-plane magnetic fields, particularly in the hybrid system without a dielectric layer. This hints at non-trivial interactions between the superconducting and ferromagnetic components of our hybrid systems when they are electronically coupled. Theoretical studies^32^,^33^ have suggested the possibility of generation of spin-triplet superconductivity at S–F interfaces in vortex-like ferromagnetic structures which stimulates further experimental studies in such hybrid structures.

## Conclusions

Two hybrid superconductor-ferromagnet systems have been investigated. In the first one the ferromagnet and superconductor are in electronic contact and vortices (antivortices) became pinned on each dark (bright) lobe of the cloverleaf-like remanent stray fields of the disks. In the second one there is an intermediate dielectric layer and only one vortex (antivortex) nucleated at the center of each disk and appeared to exhibit a surprising form of pairwise attraction with nearby interstitial vortices of the same sign. Different vortex symmetries have also been observed in the two systems. In the first structure the orientation of the cloverleaf-like stray fields strongly influences the interstitial vortex (antivortex) arrangements, completely destroying the expected triangular vortex symmetry for Nb films. In the second structure the dielectric layer eliminates the local exchange-mediated suppression of superconductivity above the ferromagnetic disk and pinning is significantly weaker. Magnetic dipole structures formed in the ‘onion’ state nucleate vortices and antivortices on the positive and negative poles respectively. An additional out-of-plane field leads to the nucleation of vortices (antivortices) and annihilation of antivortices (vortices) on the disk poles in a complex fashion. After saturation of the pinning potential on the poles interstitial vortices (antivortices) nucleate, and surprisingly this occurs near the poles of the same sign in both systems. Most of our results agree qualitatively with existing theoretical predictions, but there are suggestions of non-trivial interactions in the vortex behaviour that do not. This motivates further studies of superconductor-ferromagnetic systems with the goal of exploiting reconfigurable vortex pinning in novel superconducting devices as well as vortex imaging in systems where novel superconducting correlations can a rise such as odd frequency triplet superconductivity^11^,^12^,^13^. Furthermore, the reconfigurable vortex pinning can possibly find applications in superconducting single-photon detectors^34^ allowing a fast reset after a latch, in memory devices where one can switch between a non-dissipative and dissipative configuration, and also yields new insights into fluxonic devices.

## Additional Information

**How to cite this article:** Marchiori, E. *et al*. Reconfigurable superconducting vortex pinning potential for magnetic disks in hybrid structures. *Sci. Rep.* **7**, 45182; doi: 10.1038/srep45182 (2017).

**Publisher's note:** Springer Nature remains neutral with regard to jurisdictional claims in published maps and institutional affiliations.
